# The absence of Neuroligin-1 shapes wake/sleep architecture, rhythmic and arrhythmic activities of the electrocorticogram in female mice

**DOI:** 10.1186/s13041-025-01186-x

**Published:** 2025-04-23

**Authors:** Cassandra C. Areal, Nicolas Lemmetti, Tanya Leduc, Clément Bourguignon, Jean-Marc Lina, Erika Bélanger-Nelson, Valérie Mongrain

**Affiliations:** 1 Department of Medicine, Université de Sherbrooke, Sherbrooke, Canada; 2 Center for Advanced Research in Sleep Medicine, Recherche CIUSSS-NIM, Montréal, Canada; 3 Department of Neuroscience, Université de Montréal, Montréal, Canada; 4 Centre de recherche du Centre Hospitalier de l’Université de Montréal, 900 Saint-Denis Street, Montréal, H2X 0A9 Canada; 5 Centre de recherches mathématiques, Université de Montréal, Montréal, Canada; 6 École de technologie supérieure, Montréal, Canada

**Keywords:** Synaptic adhesion protein, Sleep regulation, Electrocorticographic activity, Paradoxical sleep, Slow wave sleep, Sleep fragmentation, Sleep deprivation, Multifractal activity, Mouse

## Abstract

Associated to glutamatergic neurotransmission, Neuroligin-1 (NLGN1) is a synaptic adhesion molecule with roles in the regulation of behavioral states and cognitive function. It was shown to shape electrocorticographic (ECoG) activity during wakefulness and sleep in male mice, including aperiodic activity under baseline conditions. Given that the expression of *Neuroligins* (*Nlgn*) differs between sexes, we here aim to characterize the impact of the absence of NLGN1 on the wakefulness and sleep architecture, rhythmic and arrhythmic activity dynamics, and responses to sleep deprivation in female animals. *Nlgn1* knockout (KO) female mice and wild-type (WT) female littermates were implanted with ECoG electrodes, and ECoG signals were recorded for 48 hours comprising a 24-hour baseline, followed by a 6-hour sleep deprivation and 18 hours of undisturbed recovery (REC). Time spent in wakefulness, slow wave sleep (SWS) and paradoxical sleep (PS), and their alternation were interrogated, and ECoG activities were quantified using a standard spectral analysis and a multifractal analysis. *Nlgn1* KO females spent more time in PS during the light period under baseline in comparison to WT females. This difference was observed along with more PS bouts and a shorter overall PS bout duration, indicative of a fragmented PS. Additionally, *Nlgn1* KO females displayed less ECoG power between 8 and 13 Hz during wake, less power between 1.25 and 3.5 Hz during PS, and more between 2.5 and 3.75 Hz during SWS in comparison to WT. Under both baseline and REC, NLGN1 absence in females was significantly associated with a higher value of the most prevalent Hurst exponent (*Hm*) during SWS, which points to a higher persistence across scales of ECoG aperiodic activity. Indications for alterations in the daily dynamics of the *Dispersion* of Hurst exponents around *Hm* were also found during SWS in KO females. The present study highlights differences in wake/sleep architecture, and in periodic (rhythmic) and aperiodic (arrhythmic/multifractal) activities in female mice lacking NLGN1. These findings provide additional support to a role for NLGN1 in shaping the ECoG organization, in particular during sleep, and will help understanding the origin of sleep disturbances in neuropsychiatric diseases.

## Introduction

Neuroligins (NLGNs) are adhesion proteins mainly found at postsynaptic sites in the mammalian brain, where they interact with presynaptic partners neurexins [1]. Four *Nlgn* genes have been identified in rodents (*Nlgn1-4*) [2, 3], and five *NLGN* genes in humans (*NLGN1* and *2* from autosomes, *NLGN3* and *NLGN4X* from the X chromosome and *NLGN4Y* from the Y chromosome; [4–8]). NLGN1 is found mainly at glutamatergic synapses and is required for adequate excitatory synaptic transmission, the generation of LTP notably in the hippocampus, and the regulation of N-methyl-D-aspartate (NMDA) receptors [9–14]. NLGN1 was shown to be dysregulated in neurodevelopmental and neurodegenerative conditions, like autism spectrum disorders and Alzheimer’s disease [15–17], both characterized by sleep disturbances [18–20].

Wakefulness/sleep architecture and electrocorticographic (ECoG) activity have been shown to be altered in animals with genetic modifications of NLGNs. For instance, a missense (gain-of-function) mutation of NLGN3 (R451C) was shown to decrease delta power during slow wave sleep (SWS; also known as non-rapid eye movement sleep) in male mice [21], whereas the absence of NLGN3 in male rats was reported to decrease SWS amount and to increase SWS delta power [22]. In addition, we previously observed that mice lacking NLGN2, which is predominantly found at inhibitory synapses [23], spend less time in SWS and have an increased consolidation of wakefulness and sleep under undisturbed and high sleep need conditions [24, 25]. Concerning NLGN1 specifically, we have shown that *Nlgn1* knockout (KO) male mice spend less time awake during the dark period and express higher delta activity during SWS particularly after sleep deprivation [26, 27]. Moreover, male mice lacking NLGN1 showed alterations in ECoG multifractal patterns, which have been linked to memory processing and object recognition speed [28, 29], with more anti-persistence during wakefulness (less self-similarity across time scales) [30]. However, the effects of the absence of NLGN1 on wakefulness/sleep amount and ECoG activity remain to be established in females.

In humans and rodents, sex differences in sleep phenotypes have been repeatedly described [31–38]. Moreover, differences between males and females in the impact of a genetic manipulation on wake/sleep variables were reported in rodents. For instance, the absence of the transcription factor neuronal Per-Arnt-Sim domain protein 2 (NPAS2) was observed to decrease time spent in paradoxical sleep (PS; also known as rapid eye movement sleep) and to reduce SWS rebound after sleep deprivation only in male mice [39]. Given that genes coding for some NLGNs are on sex chromosomes [1, 3], and that the expression of the *Nlgn* genes and NLGN protein levels were shown to be influenced by sex hormones, including in neurons [40–44], it is particularly important to verify whether the genetic manipulation of *Nlgn1* also impacts the regulation of wakefulness and sleep in females.

In the present study, we are testing the hypothesis that the absence of NLGN1 will modify wakefulness/sleep architecture and ECoG rhythmic and multifractal/arrhythmic activities in female mice. The ECoG of female *Nlgn1* KO mice and wild-type (WT) littermates has been recorded under undisturbed/normal conditions and subsequently following sleep deprivation to investigate the response to a sleep homeostatic challenge. We reveal that female mice lacking NLGN1 spend more time in PS and have a less consolidated PS, have rhythmic activity alterations in multiple frequency bands during wake and sleep states, and show more persistence across different scales of the ECoG signal during SWS. These observations provide further support for a role of NLGN1 in the regulation of sleep and underscore the relevance of this relationship also in females.

## Results

### More time spent in PS in *Nlgn1* KO females

**Fig. 1:**
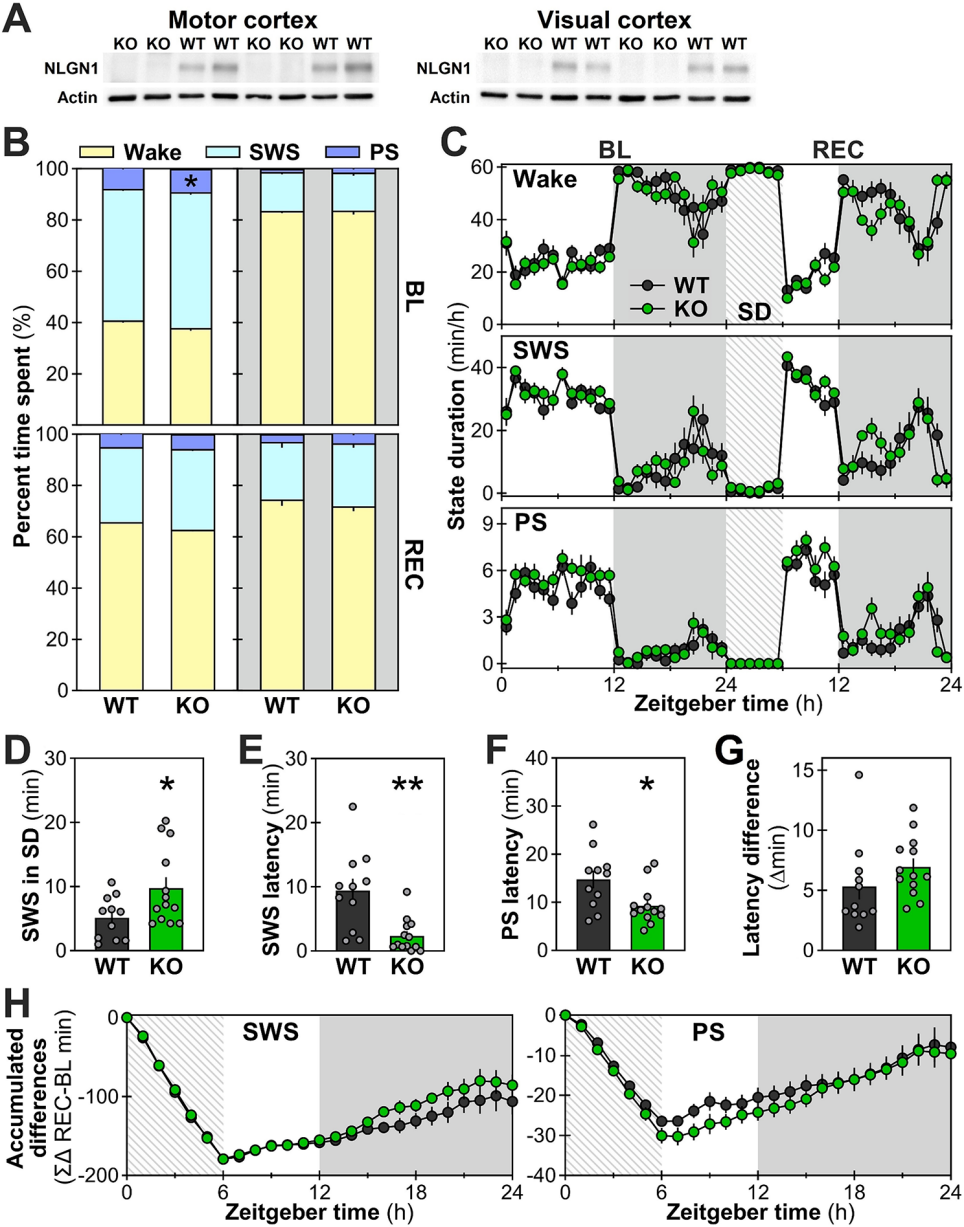
Time spent in wakefulness and sleep states in female *Nlgn1* KO and WT littermates. **A.** Western blots showing the absence of NLGN1 protein detected in the cerebral cortex regions targeted by the electrocorticographic electrodes. **B.** Proportion of the 12-hour light and dark periods occupied by wakefulness, slow wave sleep (SWS) and paradoxical sleep (PS) during baseline (BL, upper panel) and sleep deprivation (SD) + recovery (REC, lower panel). For BL, a Genotype-by-Period interaction was found for PS with a significant genotype difference during the light period (F_1,22_ = 4.6, *p* = 0.04; *: difference from the corresponding value in WT mice *p* = 0.02). No significant interaction or genotype effect was found for wake and SWS under BL (Genotype-by-Period interactions F_1,22_ ≤ 1.6, *p* ≥ 0.2; main Genotype effects F_1,22_ < 2.0, *p* > 0.1) and for all states during REC (Genotype-by-Period interactions F_1,22_ < 0.2, *p* ≥ 0.7; main Genotype effects F_1,22_ ≤ 4.2, *p* > 0.05). WT: *n* = 11, KO: *n* = 13 (also for all following panels). **C.** State duration per hour in WT and KO mice. No Genotype-by-Hour interaction was found for any of the three states during BL (F_23,506_ < 1.4, *p* > 0.1) and REC (F_17,374_ < 1.6, *p* > 0.1). Under BL and REC, no genotype effect was observed for wake and SWS (F_1,22_ < 2.9, *p* ≥ 0.1), as well as for PS (F_1,22_ ≤ 4.3, *p* ≥ 0.05). **D.** SWS measured during the 6-hour SD. *Nlgn1* KO mice had significantly more SWS than WT mice during SD (t = -2.3, *p* = 0.03). **E.** SWS latency after SD (significantly shorter in KO compared to WT mice; t = 3.7, *p* = 0.004). **F.** PS latency after SD (significantly shorter in KO compared to WT mice; t = 2.6, *p* = 0.02). **G.** Latency difference between SWS and PS did not significantly differ between WT and KO mice (t = -1.3, *p* = 0.2). **H.** Accumulated differences between the 24-hour REC and the 24-hour BL calculated for SWS (left panel) and PS (right panel). No significant Genotype-by-Hour interaction was found (F_23,506_ < 1.3, *p* > 0.2), and no significant main Genotype effect (F_1,22_ < 1.3, *p* > 0.2). Gray backgrounds indicate the 12-hour dark periods. Hatched areas represent the 6-hour SD

To verify the implication of NLGN1 in shaping wakefulness and sleep architecture in female mice, vigilance states were recorded for 24 hours under undisturbed conditions (baseline) in *Nlgn1* KO and WT littermates. Next, at the beginning of the second day of recording, a 6-hour sleep deprivation was performed by gentle handling followed by 18 hours of undisturbed/recovery conditions (sleep deprivation + recovery [REC]) to explore the role of NLGN1 in the responses to sleep loss. The absence of NLGN1 protein for the recorded cortical areas in KO mice was confirmed in a subsample of animals (Fig. 1A). During baseline, no changes were observed for time spent in wakefulness and SWS. However, the lack of NLGN1 increased the time spent in PS during the baseline light period (Fig. 1B, upper panel). Indeed, *Nlgn1* KO mice spent ~ 9 more minutes (65.8 minutes) in PS compared to WT mice (56.6 minutes), representing 16.2% more PS. This difference was restricted to the 12-hour light period (i.e., not significant for the 12-hour dark period). During the following 24 hours (REC), *Nlgn1* KO females were not significantly different from WT females for the time spent in wakefulness, SWS and PS (applicable to light and dark periods; Fig. 1B, lower panel). Furthermore, the 24-hour baseline and 24-hour REC time courses of hourly sleep/wake values revealed no overall genotype differences in any of the three vigilance states (tendency for genotypes to differ for PS during both baseline and REC: *p* = 0.05; Fig. 1C). The increased time spent in PS is thus not linked to changes at specific hours, but rather reflects a global increase over the baseline light period.

During sleep deprivation, female *Nlgn1* KO mice were strikingly difficult to maintain awake. Indeed, KO mice had around 5 more minutes of SWS during the 6-hour sleep deprivation, which is almost two times more SWS than WT mice (Fig. 1D; no PS during sleep deprivation for both genotypes as shown in the bottom panel of Fig. 1C). In line with this, *Nlgn1* KO mice fell asleep significantly faster, with both SWS and PS reached sooner than WT littermates after the end of the sleep deprivation (i.e., shorter latencies; KO taking a fourth of the WT time to reach SWS, and two-thirds of the WT time to reach PS; Fig. 1E and G). However, the global loss of SWS and PS as well as the general recovery of these states after sleep deprivation as assessed using accumulated differences from baseline was not significantly impacted by the *Nlgn1* mutation (Fig. 1H). Thus, the absence of NLGN1 in female mice increased SWS during sleep deprivation and decreased the latencies to sleep states after the end of sleep deprivation.

### Fragmented states after sleep deprivation in *Nlgn1* KO females

**Fig. 2:**
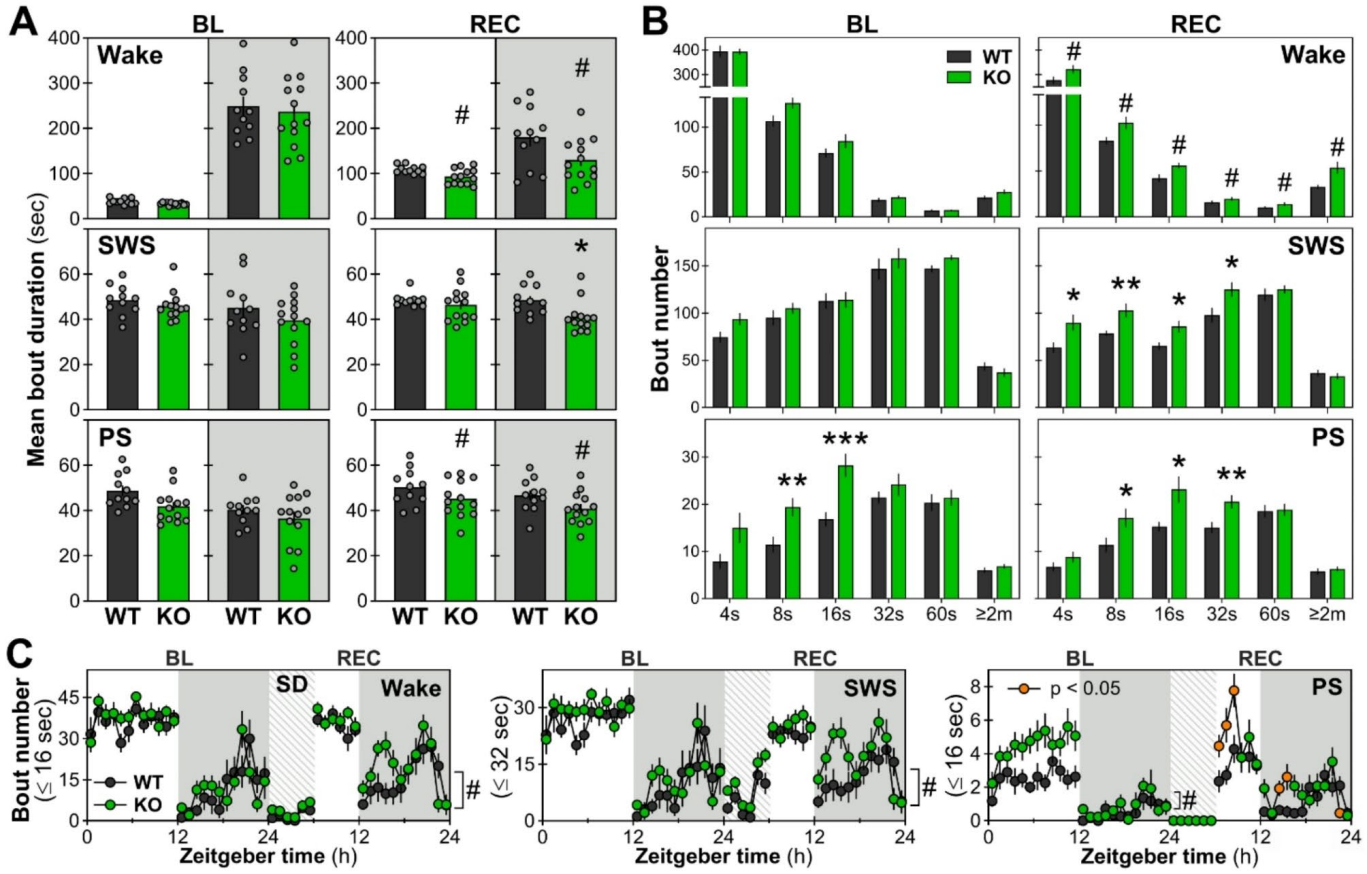
Wake/sleep fragmentation in *Nlgn1* KO and WT female mice for baseline (BL) and recovery (REC). **A.** Mean duration of individual bouts in seconds for BL and REC, depicted separately for the light and dark periods. For BL, no significant Genotype-by-Period interaction was found for wake, SWS, and PS (F_1,22_ < 0.6, *p* > 0.4) and no genotype effect for any of the states (F_1,22_ < 4.1, *p* > 0.05). For REC, a significant Genotype-by-Period interaction was found for SWS (F_1,22_ = 4.4, *p* < 0.05; *: *p* < 0.05 relative to the corresponding value in WT mice, also in panel B), and significant genotype effects were found for wakefulness and PS (#: F_1,22_ > 5.7, *p* < 0.03). WT: *n* = 11, KO: *n* = 13 (also for all following panels). **B.** Bout number of different durations in seconds or minutes for BL and REC. During BL, a significant Genotype-by-Duration interaction was found for PS (F_5,110_ = 3.2, *p* = 0.01; **: *p* < 0.01 and ***: *p* ≤ 0.001 from the corresponding value in WT mice). No interaction or genotype effects was found in the number of bouts for wake and SWS during BL (F_1/5,22/110_ < 2.2, *p* > 0.1). During REC, significant Genotype-by-Duration interactions were found for SWS and PS (F_5,110_ > 2.8, *p* ≤ 0.04). A significant genotype effect was found for the number of bouts during wakefulness (#: F_1,22_ = 12.7, *p* = 0.002). **C.** BL and REC time courses of the number of brief awakening (≤ 16 seconds), short SWS (≤ 32 seconds) and PS bouts (≤ 16 seconds). During BL, no significant interaction was found for any of the states (F_23,506_ ≤ 1.6, *p* > 0.05). A significant genotype effect was found for PS (#: F_1,22_ = 13.0, *p* = 0.002), but not for wake and SWS (F_1,22_ < 1.4, *p* > 0.2). During REC, a significant Genotype-by-Hour interaction was found for PS (F_17,374_ = 2.2, *p* = 0.009), and significant genotype effects were found for wake and SWS (#: F_1,22_ > 7.6, *p* < 0.02). Orange datapoints for KO mice indicate hours with significant genotype differences (*t*-test *p* < 0.05). Gray backgrounds indicate the 12-hour dark periods. Hatched areas represent the 6-hour sleep deprivation (SD)

To assess wake/sleep fragmentation/consolidation, the mean duration of individual bouts of the three states were compared between genotypes together with the number of bouts of different durations. For bout duration, female mice lacking NLGN1 had generally shorter wake/sleep bouts, which reached statistical significance only during REC (only REC dark period for SWS; shorter of approximately 10 to 30%; Fig. 2A). Additionally, *Nlgn1* KO female mice displayed a general increase in the number of wake bouts of both short and long durations and in the number of short SWS bouts during REC in comparison to WT female littermates (Fig. 2B, upper and middle panels). For PS, *Nlgn1* KO mice showed an increased number of relatively short (i.e. ≤ 32-second) bouts during baseline and REC in comparison to WT mice (Fig. 2B, lower panels).

Bout number of specific durations were computed for each recording hour for the three vigilance states to investigate potential genotype-dependent differences in their daily distribution. For wakefulness, bouts ≤ 16 seconds have been considered as brief awakenings and used as an indication of state discontinuity [45, 46]. For SWS, bouts ≤ 32 seconds were quantified, as a 60-second duration bout is considered indicative of consolidated bouts [47]. *Nlgn1* KO female mice showed more short wake and SWS bouts than WT female littermates only during REC (Fig. 2C, left and middle panels). Moreover, KO mice had more short PS bouts; a difference applicable to baseline, to the first hours after sleep deprivation during the light period and to the beginning of the dark period during REC (Fig. 2C, right panel).

Overall, the increased number of shorter PS bouts paired with a reduced duration of individual bouts suggests a fragmented PS in *Nlgn1* KO females. Thus, despite an increase in total PS time, the observed fragmentation suggests an instability of the state. Interestingly, there was also a significantly higher number of transitions from SWS to PS in females *Nlgn1* KO mice in comparison to WT littermates for both the baseline and REC conditions (BL: WT = 79.0 ± 1.9, KO = 108.7 ± 7.8, *p* < 0.01; REC: WT = 67.8 ± 3.5, KO = 88.0 ± 5.7, *p* = 0.01), which likely contributes to both the elevated number of short PS bouts and increased time spent in PS. Additionally, the absence of NLGN1 interferes with wake and SWS consolidation in female mice, significantly during sleep deprivation and the recovery period, as observed by the reduction of bout duration and the increase in the number of shorter SWS bouts.

### Altered ECoG rhythmic activity in *Nlgn1* KO females

**Fig. 3:**
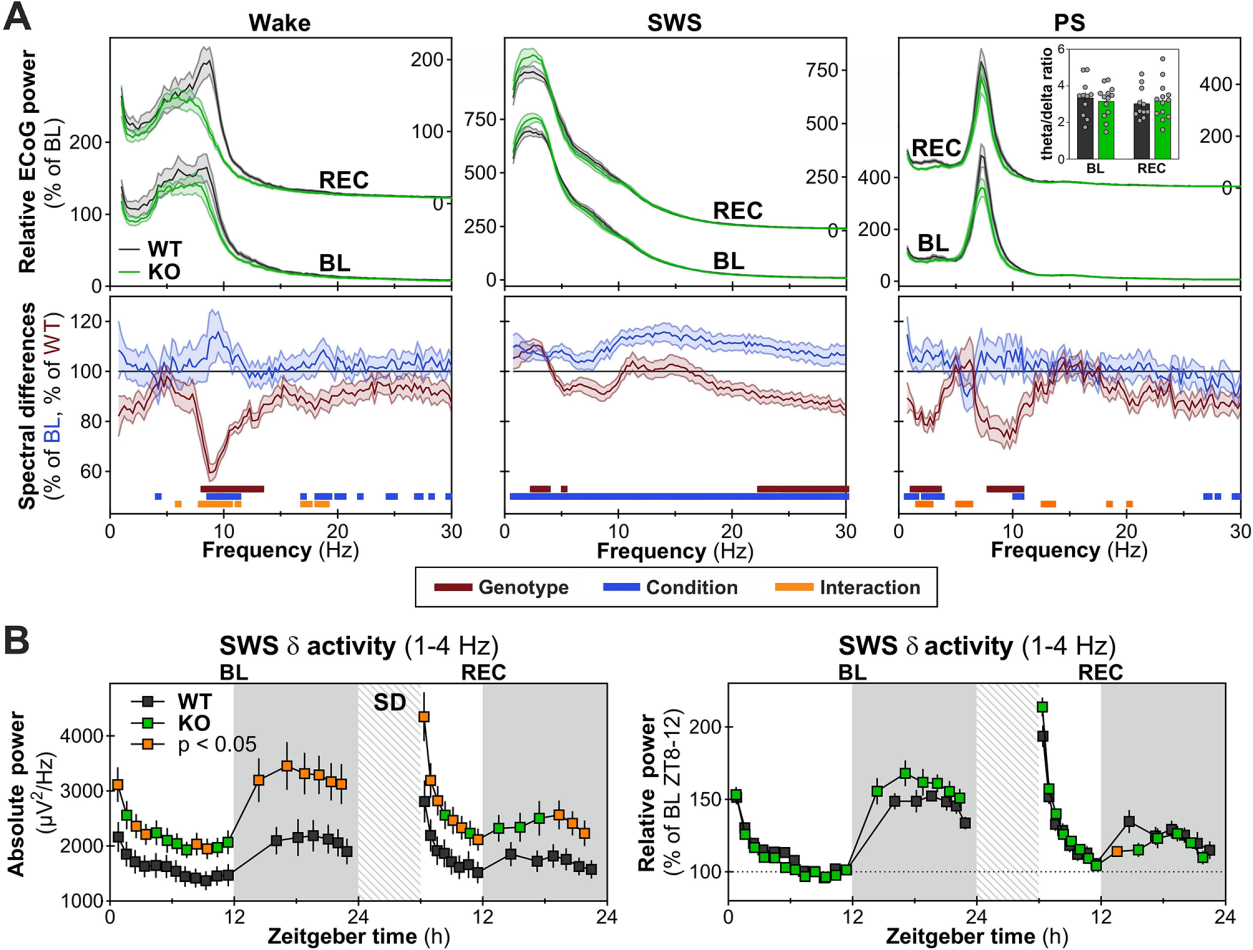
ECoG spectral power under baseline (BL) and recovery (REC) conditions for wakefulness, slow wave sleep (SWS) and paradoxical sleep (PS). **A.** Upper panels: relative power with the left y-axes used for BL and right y-axes for REC. Lower panels: spectral power differences expressed, for KO mice (red line; *n* = 13 means of BL-REC) as percent WT mice (= 100%; *n* = 11 means of BL-REC), and for REC (blue line; *n* = 24 KO and WT mice) as percent of BL (= 100%; *n* = 24). Red and blue bars above the x-axes indicate, respectively, significant genotype and condition main effects (F_1,22_ > 4.3, *p* < 0.05), and orange bars significant Genotype-by-Condition interactions (F_1,22_ > 4.4, *p* < 0.05). The inset in upper right panel shows the ratio of theta (6-9 Hz) to delta (1-4 Hz) activity in PS (Genotype-by-Condition interaction F_1,22_ = 3.1, *p* = 0.09; main Genotype effect F_1,22_ = 0.0, *p* = 1.0). **B.** Time course of absolute (left panel) and relative (right panel) SWS delta (δ; 1-4 Hz) activity in *Nlgn1* KO and WT mice during BL and REC. For absolute δ activity, significant Genotype-by-Interval interactions were found for BL (F_17,374_ = 4.2, *p* = 0.008) and REC (F_13,286_ = 4.5, *p* = 0.003). For relative δ activity, a significant interaction was found for REC only (F_13,286_ = 2.8, *p* = 0.01). Orange datapoints for KO mice indicate intervals with significant genotype differences (*t*-test *p* < 0.05). WT: *n* = 11, KO: *n* = 13. Gray backgrounds indicate the 12-hour dark periods. Hatched areas represent the 6-hour sleep deprivation (SD)

To examine the role of NLGN1 in shaping the quality of wake/sleep states in female mice, the ECoG power spectrum (shown for frequencies between 0.75 and 30 Hz) was compared between genotypes for the three vigilance states separately for baseline and REC. During wakefulness, *Nlgn1* KO mice showed less activity from 8 to 13 Hz, encompassing high theta and alpha range. This difference was more prominent during REC for most Hz-bins (Fig. 3A, left column). As expected, spectral power during SWS was increased in REC compared to baseline for all frequencies (Fig. 3A, middle column). In addition, when considering baseline and REC recordings together, female mice lacking NLGN1 expressed more delta activity (between 2.5 and 3.75 Hz), and less beta activity (> 22 Hz) during SWS (Fig. 3A, middle column). During PS, KO mice showed less ECoG activity in the delta range (between 1.25 and 3.5 Hz), particularly during REC, and less activity between 8 and 10.75 Hz in baseline and REC (Fig. 3A, right column). Nevertheless, the ratio of activity between theta and delta during PS was not significantly impacted by the *Nlgn1* mutation (Fig. 3A, inset of upper right panel). Overall, *Nlgn1* KO female mice display alterations in spectral ECoG activity in all three vigilance states, with state-specific signatures that also depend on the experimental condition (i.e., baseline versus REC).

**Fig. 4:**
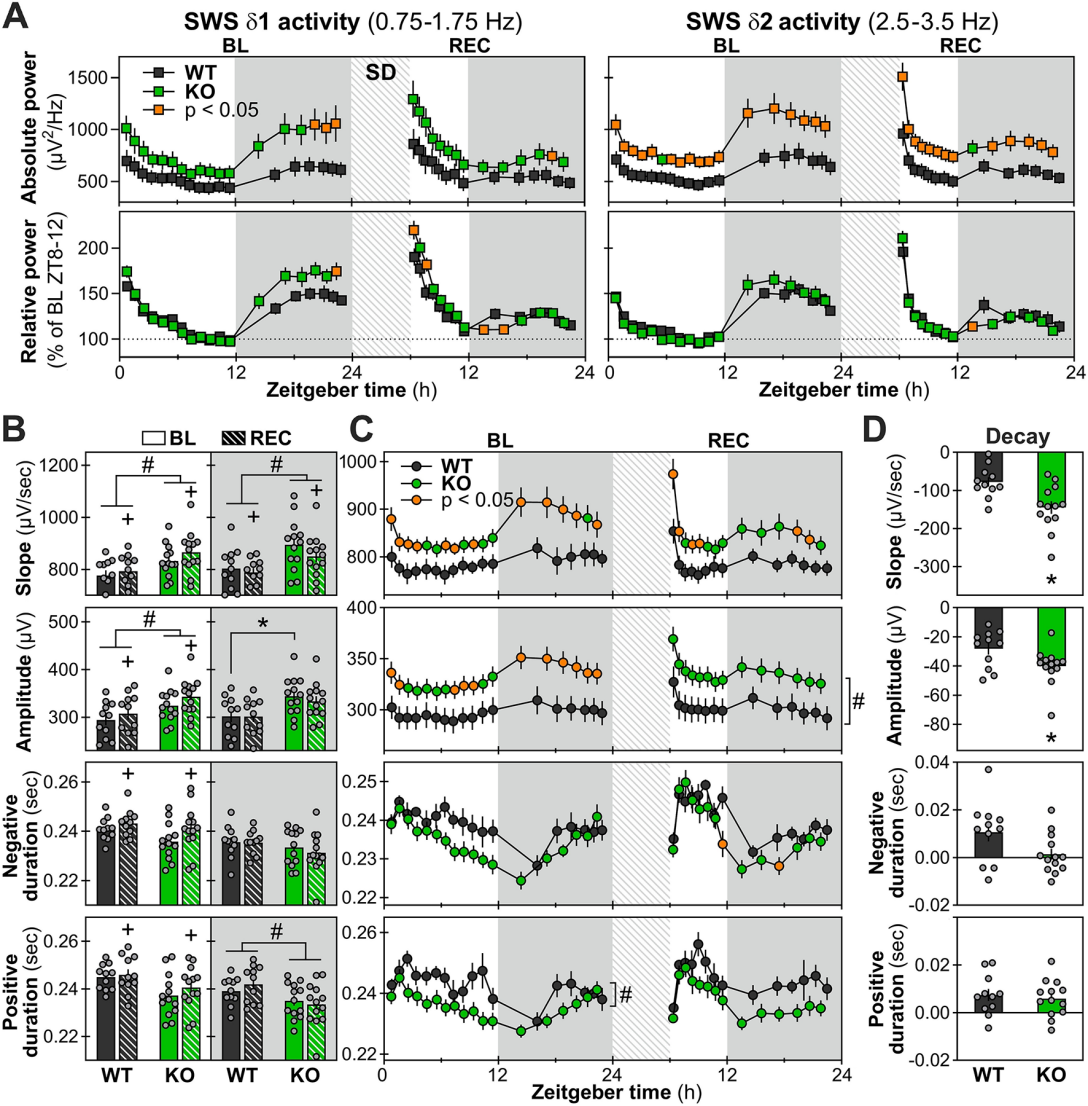
Time courses of SWS spectral activity in delta (δ) sub-bands and properties of slow waves. **A.** Time courses of absolute (upper panels) and relative (lower panels) SWS δ1 (0.75-1.75 Hz) and δ2 (2.5-3.5 Hz) activity in female *Nlgn1* KO and WT mice during baseline (BL) and recovery (REC). For absolute δ1 and δ2 activity, significant Genotype-by-Interval interactions were found for BL (F_17,374_ > 3.1, *p* < 0.03) and REC (F_13,286_ > 3.2, *p* < 0.03). For relative activity during BL, a significant Genotype-by-Interval interaction was found for δ1 only (F_17,374_ = 2.9, *p* = 0.007), and no interaction or genotype effect was found for δ2 (F_1/17,22/374_ < 1.2, *p* > 0.2). During REC, significant interactions were found for both relative δ1 and δ2 activity (F_13,286_ > 2.0, *p* < 0.05). Orange datapoints for KO mice indicate intervals with significant genotype differences (*t*-test *p* < 0.05; also in panel C). WT: *n* = 11, KO: *n* = 13 (also for all following panels). **B.** Slow wave (SW) properties (slope, amplitude, negative duration, positive duration) averaged during light and dark periods for BL and REC. For light periods, no significant Genotype-by-Condition interaction was found for any of the parameters (F_1,22_ ≤ 3.8, *p* > 0.06), but significant genotype effects were found for slope and amplitude (#: F_1,22_ > 4.4, *p* < 0.05; negative and positive durations: F_1,22_ < 4.3, *p* > 0.05). Also, significant condition effects were found for all parameters (+: F_1,22_ > 4.4, *p* < 0.05). For dark periods, a significant Genotype-by-Condition interaction was found for amplitude (F_1,22_ = 9.6, *p* = 0.005; *: *p* < 0.05 relative to the corresponding value in WT mice; interaction for slope, negative and positive durations: F_1,22_ < 3.8, *p* > 0.06), significant genotype effects were found for slope and positive duration (#: F_1,22_ > 4.8, *p* < 0.04; genotype effect for negative duration: F_1,22_ = 1.6, *p* = 0.2), and a significant condition effect for slope (+: F_1,22_ = 15.6, *p* = 0.0007; negative and positive durations: F_1,22_ < 0.8, *p* > 0.3). **C.** BL and REC time courses of SW properties averaged across intervals with the same number of SWS epochs. During BL, significant Genotype-by-Interval interactions were observed for SW slope and amplitude (F_17,374_ ≥ 3.6, *p* ≤ 0.005; negative and positive duration: F_17,374_ < 1.7, *p* > 0.05), and a significant genotype effect was found for positive duration (#: F_1,22_ = 6.1, *p* = 0.02; negative duration: F_1,22_ = 3.5, *p* = 0.08). During REC, significant interactions were observed for SW slope and negative duration (F_13,286_ > 2.1, *p* < 0.03; amplitude and positive duration: F_13,286_ < 1.9, *p* > 0.07), and a significant genotype effect for amplitude (#: F_1,22_ = 4.5, *p* < 0.05; positive duration: F_1,22_ = 3.8, *p* = 0.06). **D.** Decay of SW properties between the first and the last interval of the light period of REC. Significant differences were observed for SW slope and amplitude (t > 2.2, *p* < 0.04), but not for negative and positive durations (t < 2.1, *p* ≥ 0.06). Gray backgrounds indicate the 12-hour dark periods. Hatched areas represent the 6-hour sleep deprivation (SD)

Next, the time course of SWS delta activity (1-4 Hz) was analyzed using both absolute and relative activity separately for baseline and REC. When compared to WT female littermates, absolute delta activity was significantly higher at specific hours during baseline and REC in *Nlgn1* KO females (Fig. 3B, left panel). However, the time course of relative delta activity, which is more reliable to unveil differences in individual dynamics given that it takes into account differences in absolute power [47, 48], did not differ between KO and WT females during baseline, and was only impacted by genotype during REC, with KO mice showing a lower activity for the first interval of the dark period (Fig. 3B, right panel). Given that different subdivisions of delta activity (delta 1 = 0.75-1.75 Hz and delta 2 = 2.5-3.5 Hz) were shown to have different dynamics in response to prior sleep-wake history [49], the time courses of activity in these smaller bands were compared between female *Nlgn1* KO and WT mice. Absolute delta 1 activity was significantly higher in KO compared to WT mice specifically at the end of the dark periods for both baseline and REC (Fig. 4A, upper left panel), while absolute delta 2 activity was generally higher in KO females throughout both recording conditions (some time intervals reaching an ∼60% increase compared to WT; Fig. 4A, upper right panel). Relative delta 1 activity was also significantly higher in KO than WT mice but specifically at the end of baseline and immediately after sleep deprivation, whereas it was lower in KO mice at the beginning of the dark period in REC (Fig. 4A, bottom left panel). Relative delta 2 activity was significantly lower in KO compared to WT mice only for the first interval of the dark period in REC (Fig. 4A, bottom right panel). In general, although the absence of NLGN1 in female mice appears to impact absolute delta 2 activity to a greater extend than absolute delta 1 activity, an opposite observation is made when considering the dynamics of relative delta activity in these smaller bands.

### Higher amplitude and slope of slow waves in *Nlgn1* KO females

Some properties of slow waves (SW) during SWS can represent additional markers of sleep intensity, especially when considering the slope and amplitude of SW that are higher under conditions of elevated homeostatic sleep pressure [27, 50, 51]. We indeed found here that SW slope, amplitude and duration were increased in REC compared to baseline when considering the light period during which recovery occurs (Fig. 4B). The slope of SWS SW was found to be significantly steeper in female *Nlgn1* KO mice when compared to female WT littermates, independently of the light/dark periods and of baseline/REC conditions (Fig. 4B, first row). This difference was particularly prominent at the beginning of the light and dark periods during baseline and immediately after sleep deprivation during REC (steeper of 10% or more in comparison to WT; Fig. 4C, first row), moments at which homeostatic sleep pressure is highest. The amplitude of SW was observed to be significantly higher (∼10%) in *Nlgn1* KO females in comparison to WT females during light periods (baseline and REC), and for the baseline dark period (Fig. 4B, second row). When considering the 24-hour dynamics of SW amplitude, it was modified in the absence of NLGN1 only for baseline (Fig. 4C, second row), with KO mice showing higher amplitude at specific times during the light period and for all intervals of the dark period, whereas SW amplitude was constantly higher in KO (independent of interval) during REC. Female *Nlgn1* KO mice also showed a significantly shorter duration of the positive peak of SW, indicative of the ON phase of neuronal firing [52], than WT animals during dark periods (Fig. 4B; fourth row), which was also observed when considering the 24-hour dynamics during baseline (Fig. 4C, fourth row). The duration of the negative peak of SW, indicative of the OFF phase of neuronal firing [52], was found to be decreased in *Nlgn1* KO mice at specific intervals during REC when compared to WT littermates (Fig. 4C, third row).

To further investigate the effects of the mutation on the homeostatic-dependent dynamics of SW properties, decay values were computed as the difference between the first and the last interval of the first 6 hours following sleep deprivation. There were significantly greater decays of SW slope and SW amplitude in female *Nlgn1* KO mice when compared to female WT littermates (Fig. 4D, first and second rows). However, the decay of SW negative and positive durations was not significantly different between genotypes (Fig. 4D, third and fourth rows). These observations reveal that the absence of NLGN1 in female mice drives multiple alterations in SW properties during SWS, in addition to modifying their 24-hour dynamics under undisturbed and high homeostatic sleep pressure conditions.

### Higher persistence in SWS arrhythmic activity in *Nlgn1* KO females

Spectral analysis of the ECoG quantifies the activity for distinct brain rhythms, but lacks information about the general organization of the signal across different time scales, something often referred to as arrhythmic or aperiodic activity. Multifractal analysis of the ECoG can be used to assess arrhythmic (aperiodic) activity of the signal and the long-range relationship in the activity across different time scales [30, 53]. To characterize the aperiodic activity of the ECoG signal in female *Nlgn1* KO mice, we quantified two parameters: the most prevalent Hurst exponent (*Hm*) and the *Dispersion* of Hurst exponents around *Hm* [30, 54]. *Hm* was used as a measure of long-range temporal correlation/dependency of ECoG oscillations. It represents an indication of “anti-persistence” of the organization of the activity across all frequencies of the interrogated spectrum (0.5 to 64 Hz), with lower *Hm* values pointing to a higher anti-persistence (less long-range dependency), and higher values to less anti-persistence (more self-similarity across scales) [30]. *Dispersion* refers to the variability of *Hm* when looking at discrete (local) points in time. It was used as an indicator of “multifractality” or “local instability”. *Dispersion* values closer to 0 are indicative of more local stability of the power decay across frequencies (i.e., less variability of *Hm* at a given timepoint, thus fewer fractal dynamics in the signal), and more negative *Dispersion* values indicative of lower local stability (i.e., more local variability of *Hm* or greater variability of fractal dynamics referred to as multifractality).

**Fig. 5:**
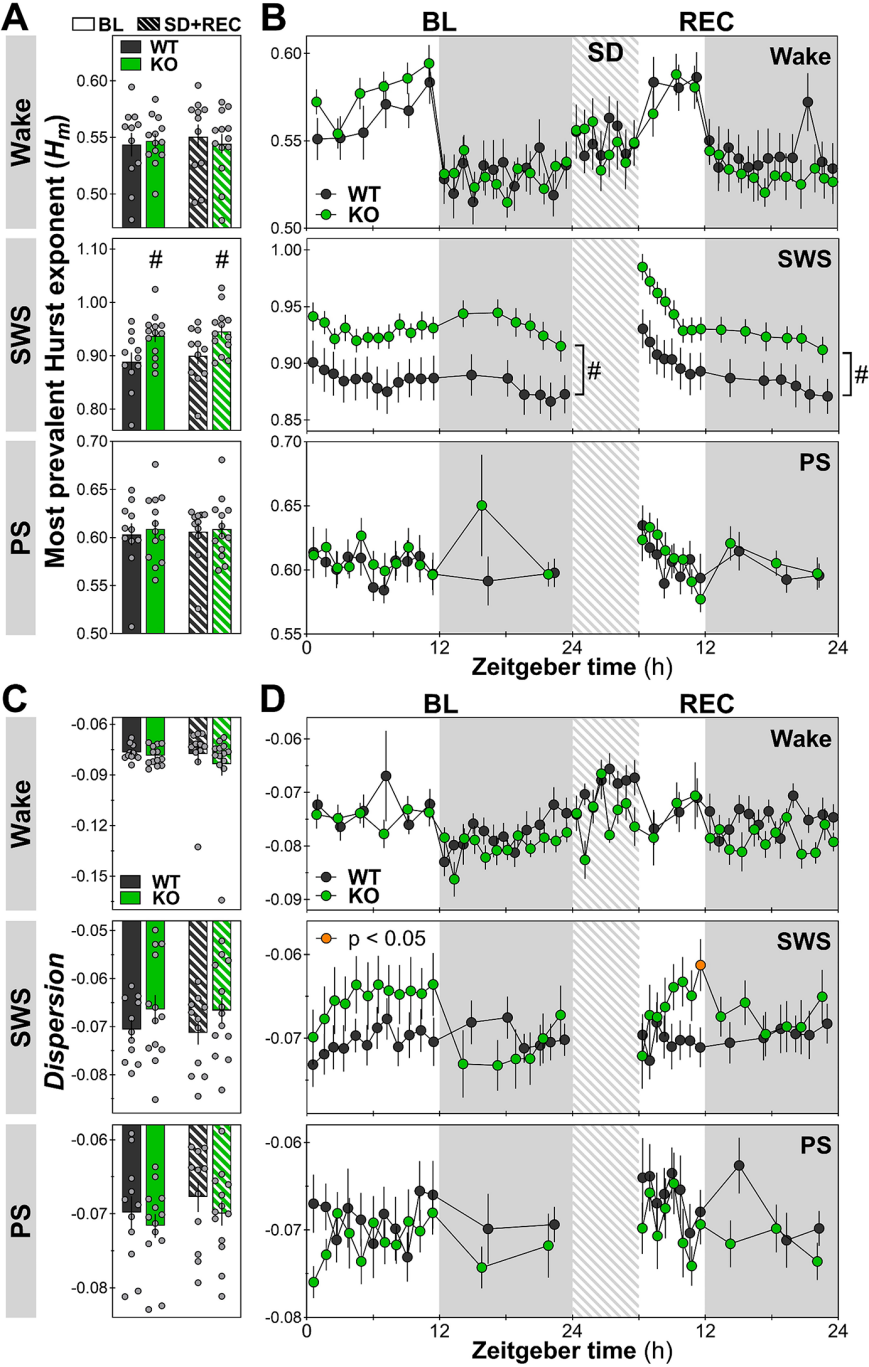
The most prevalent Hurst exponent (*Hm*) and *Dispersion* of exponents around *Hm* quantified for wakefulness, SWS, and PS during baseline (BL) and recovery (REC). **A. ***Hm* values averaged over 24 hours for BL and REC. No significant Genotype-by-Condition interaction was found for any of the three states (F_1,22_ < 2.0, *p* > 0.1), but a significant genotype effect was found for SWS (#: F_1,22_ = 5.9, *p* = 0.02; for wake and PS: F_1,22_ ≤ 0.1, *p* > 0.7). WT: *n* = 11, KO: *n* = 13 (also for panel C). **B.** 24-hour time courses of *Hm* for all vigilance states. No significant Genotype-by-Interval interaction was found for any of the states during BL and REC (F_10/12/13/17/22,200/228/260/340/440_ ≤ 1.6, *p* > 0.05), but genotype effects were observed for SWS during both BL and REC (#: F_1,20_ ≥ 5.3, *p* < 0.04; for wake and PS: F_1,20_ < 0.3, *p* ≥ 0.6). WT: *n* = 10, KO: *n* = 12 (also for panel D). **C. ***Dispersion* values averaged over 24 hours for BL and REC. No significant Genotype-by-Condition interaction was found for any of the states (F_1,22_ < 0.3, *p* > 0.6), and also no significant genotype effect was found (F_1,22_ < 1.5, *p* > 0.2). **D.** 24-hour time courses of *Dispersion* for each state. Genotype-by-Interval interactions are observed for SWS during both BL and REC (F_13/17,260/340_ ≥ 3.8, *p* < 0.0001; wake and PS during BL or REC: F_10/12/17/22,200/228/340/440_ ≤ 1.3, *p* > 0.1). No genotype effect was found for wake and PS (BL or REC: F_1,20_ ≤ 4.1, *p* > 0.05). The orange datapoint for KO mice indicates an interval with a significant genotype difference (*t*-test *p* < 0.05). Gray backgrounds indicate the 12-hour dark periods. Hatched areas represent the 6-hour sleep deprivation (SD)

For *Hm*, *Nlgn1* KO mice were found to have a higher value in comparison to WT littermates specifically for the ECoG signal of SWS (24-hour average), and not for wake and PS (Fig. 5A). This difference applied to both baseline and REC, and was global across the 24-hour days, thus not specific to any particular interval (Fig. 5B). For *Dispersion*, the lack of NLGN1 in female mice did not significantly modify its value in any of the states when considering the averages over 24 hours (Fig. 5C). However, the 24-hour time course during baseline and REC was found to differ between genotypes for SWS (Fig. 5D), and *Dispersion* was notably less negative in female KO mice compared to female WT littermates only at the end of the light period of REC (Fig. 5D). This result shows that multifractal activity of the ECoG signal of *Nlgn1* KO mice is altered only during SWS, with KO mice showing more self-persistence (increased *Hm*) and 24-hour variations in the local instability of aperiodic activity.

**Fig. 6:**
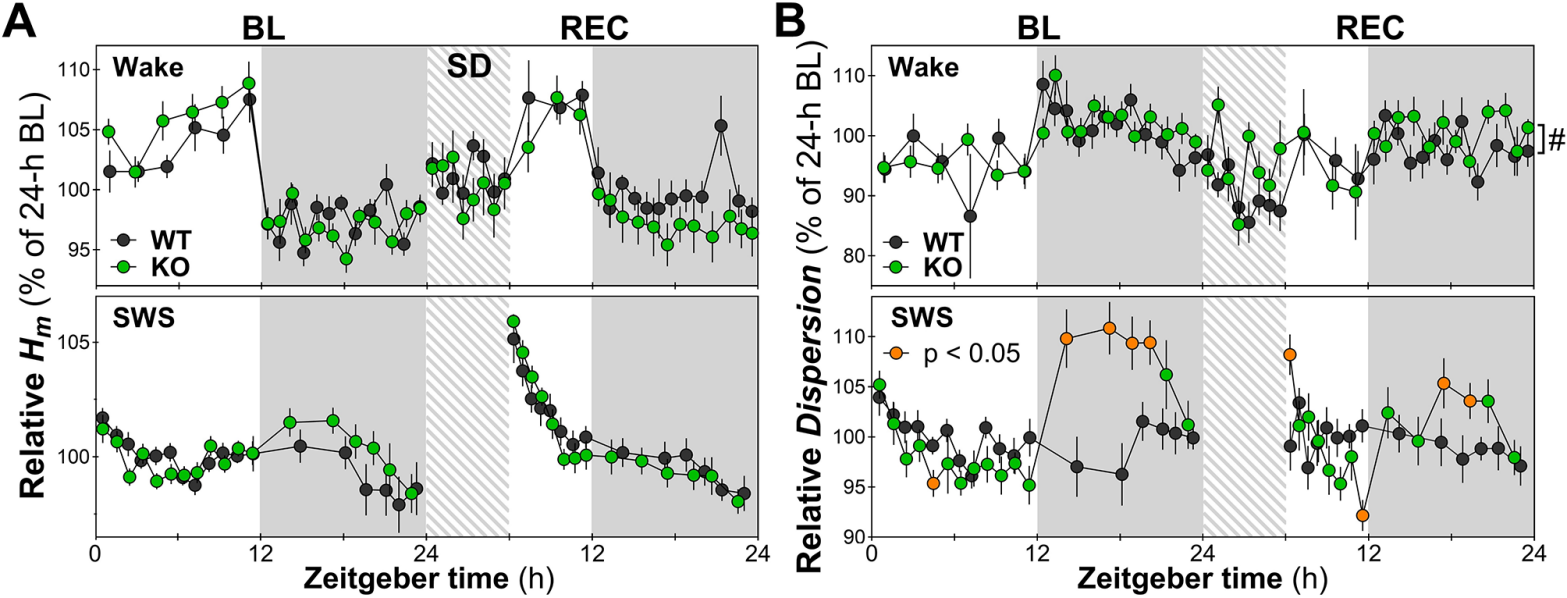
Dynamics of relative *Hm* and *Dispersion* for wakefulness and SWS during baseline (BL) and recovery (REC). **A.** Time courses of relative *Hm*. No significant Genotype-by-Interval interaction was found for wake or SWS (BL and REC: F_13/17/22,260/340/440_ ≤ 1.6, *p* > 0.07). The same applies to genotype effect (BL and REC: F_1,20_ < 2.2, *p* > 0.1). WT: *n* = 10, KO: *n* = 12 (also for panel B). **B.** Time courses of relative *Dispersion*. Significant Genotype-by-Interval interactions are observed for SWS during both BL and REC (F_13/17,260/340_ > 3.6, *p* < 0.0001). No interaction was observed for wake (F_17/22,340/440_ < 1.4, *p* > 0.1), but a genotype effect was found during REC only (#: F_1,20_ = 5.0, *p* = 0.04). Orange datapoints for KO mice indicate intervals with significant genotype differences (*t*-test *p* < 0.05). Gray backgrounds indicate the 12-hour dark periods. Hatched areas represent the 6-hour sleep deprivation (SD)

To better capture the impact of the KO of *Nlgn1* in females on the 24-hour dynamics of variations in *Hm* and *Dispersion* during wake and SWS (states occupying most of the nychthemeron), time courses of relative values were analyzed (each interval expressed relative to the corresponding baseline 24-hour average of each mouse). For *Hm*, the 24-hour variations were not impacted by the absence of NLGN1 in female mice for both wakefulness and SWS (baseline and REC; Fig. 6A). However, female *Nlgn1* KO mice had a significantly higher value of relative *Dispersion* across all wake intervals during REC (Fig. 6B, upper panel), which suggests that the wake aperiodic ECoG activity of KO mice is more multifractal (locally unstable) than that of WT littermates, specifically under conditions of higher homeostatic sleep pressure. Moreover, KO females showed much more amplitude of 24-hour variations in relative *Dispersion* measured during SWS than WT littermates during both baseline and REC (Fig. 6B, lower panel). Indeed, the relative value of *Dispersion* increased in KO animals (to become higher than WT) specifically during the dark periods and immediately after sleep deprivation, but decreased (to lower than WT) at the end of the light period of REC. This suggests that the “multifractality/local instability” of SWS ECoG aperiodic activity of *Nlgn1* KO mice fluctuates more throughout the day than that of WT animals, with it being more multifractal/locally unstable during the dark period and following a period of prolonged wakefulness.

## Discussion

Our study highlights the impacts of the lack of NLGN1 in female mice on wake/sleep architecture, rhythmic and arrhythmic activities. More specifically, *Nlgn1* KO females spent more time in PS during baseline, and showed a shorter mean duration of individual bouts and higher number of bouts for all three vigilance states during REC. Also, KO animals had modified spectral activity in all states, notably during SWS with a considerably higher delta activity, and enhanced SW slope and amplitude. Multifractal activity was altered specifically during SWS in KO when compared to WT littermates with a higher *Hm* and fluctuating *Dispersion*. Altogether, these data support a role for NLGN1 in regulating rhythmic and arrhythmic activities in female mice in a manner that mainly differs from our previous observations in males as discussed below [26, 27, 30], and strengthens the relationship between wake/sleep regulation and cell adhesion proteins found at the synapse.

During undisturbed conditions (baseline), the time spent in PS was higher in *Nlgn1* KO females compared to WT littermates. This observation could emerge from a role of NLGN1 in shaping the functioning of the pontine neurocircuitry controlling PS generation more specifically in females because of the absence of a change in the total time spent in PS in *Nlgn1* KO males [26]. Indeed, NLGN1 is required for proper glutamatergic neurotransmission [13, 14], and is expressed in the pontine tegmentum in mice (Allen Institute mouse brain atlas), a region shown to control the time spent in PS through specific glutamatergic neurons [55]. It could thus be possible that the higher time spent in PS in KO mice results from reduced excitation by *Atoh1*-expressing glutamatergic neurons onto GABAergic cells of the lateral pontine tegmentum, which inhibits the sublaterodorsal tegmental nucleus that promotes the generation of PS [55, 56]. Inhibiting GABAergic cells of the lateral pontine tegmentum was indeed shown to increase the time spent in PS without changing time spent in SWS [55], and the loss of NLGN1 might mimic this effect by reducing the excitatory drive of *Atoh1*^*+*^ glutamatergic cells onto the lateral pontine tegmentum. In parallel, the sublaterodorsal tegmentum was shown to receive direct excitatory inputs and to be composed of glutamatergic neurons [56], making it likely that other inputs as well as outputs of this PS generator are affected in the absence of NLGN1 given that it is expressed throughout pontine structures [57]. Accordingly, investigating the role of NLGN1 in this circuit in female mice via targeted manipulation could be of relevance.

Additionally, *Nlgn1* KO females exhibit fragmented PS under both baseline and REC (i.e., shorter PS bout duration in REC, more short PS bouts in baseline and REC). This apparent instability in PS maintenance could also be the consequence of changes in the pontine circuit described above. Alternatively, it could arise from alterations of glutamatergic inputs to hypothalamic orexin neurons, a neuronal population regulating the occurrence and consolidation of PS [58, 59]. Functional glutamatergic synapses were indeed reported on orexin neurons [60], and sleep deprivation was found to impair glutamatergic transmission onto orexin neurons [61]. Therefore, the loss of NLGN1 could lead to a hypoactivation of orexin neurons contributing to less consolidated PS, which could be interesting to clarify in future research. Of note, even in the absence of change to the recovery of PS after sleep deprivation, the observations of more time spent in PS together with more shorter bouts of PS in the absence of NLGN1 may suggest alterations in the ‘homeostatic’ regulation of PS (rapid eye movement sleep) [62]. This could also suggest alterations in other hypothalamic circuits (e.g., input to neurons of the preoptic area) [63], which would also be relevant to interrogate in animals lacking NLGN1.

The quality of PS was also impacted by the KO of *Nlgn1* in female mice given the observation of less ECoG power between 1.25 and 3.5 Hz and between 8 and 10.75 Hz, differences that were not found in males lacking NLGN1 [26]. Previous studies have shown that lower theta (5-7 Hz) and higher theta (8-11 Hz) are linked to tonic and phasic PS, respectively [64, 65]. As such, lower power in the 8-10.75 Hz frequency range may suggest that the loss of NLGN1 specifically affects phasic PS (i.e., presenting features of PS such as eye movements and muscle twitches [not quantified in the present study]). The literature has highlighted the contribution of the supramammillary nucleus glutamatergic projections to the dentate gyrus in the generation of theta rhythms during PS, as their stimulation was shown to enhance PS theta power and frequency [66]. Hence, the loss of NLGN1 could impair the activation by the supramammillary nucleus, resulting in a lower theta activity compared to their WT littermates. Overall, *Nlgn1* KO females display an ensemble of PS phenotypes not previously found for males, in which the lack of NLGN1 did not impact the total time spent in PS and PS ECoG spectral power [26]. This suggests differences between males and females regarding the roles of NLGN1 in the functioning of PS regulatory circuits.

The time spent awake was not modified in *Nlgn1* KO females, which differs from the reduction previously reported in KO males [26]. Nevertheless, the duration of wake bouts was shorter and the number of wake bouts higher during the sleep deprivation and recovery 24-hour period. Shorter wake bouts were found also in *Nlgn1* mutant males [26]. In addition, female *Nlgn1* KO mice were difficult to maintain awake during sleep deprivation, as indicated by the increased time spent in SWS during sleep deprivation, and showed a much shorter latency to sleep after sleep deprivation. These observations could be associated to a role for NLGN1 in the maintenance of wakefulness episodes, as we have proposed previously for males [26]. Given the established role of hypothalamic orexin neurons in the control of sleep to wake transitions [58, 67], and the modified glutamatergic inputs to orexin neurons in the context of experimental sleep loss [61], the findings of an apparent impaired capacity to maintain wakefulness under elevated homeostatic sleep pressure in the absence of NLGN1 could also be associated with a dysfunction of glutamatergic signaling to orexin neurons. Monitoring wake/sleep alternation under the manipulation of NLGN1 specifically in orexin neurons in male and female mice will be required to verify this assumption.

In addition, loss of NLGN1 in female mice impaired the quality of wakefulness reflected by a reduction of ECoG activity in theta/alpha frequencies, most strikingly during sleep deprivation and recovery. Theta/alpha activity during wakefulness is normally increased following prolonged wake in humans and rodents [68–70]. Given that glutamatergic neurons from the medial septum are contributing to hippocampal theta activity during wake via intraseptal connections [71], the loss of NLGN1 could impact wakefulness ECoG activity by disrupting this pathway. This circuitry could be particularly vulnerable to the loss of NLGN1 in female mice, as theta/alpha activity is affected during both wakefulness and PS, while only wake was found to be significantly impacted in males lacking NLGN1 [26].

One of the most striking phenotypes found here in *Nlgn1* KO female mice concerns the increase in delta activity (absolute power) during SWS, which is particularly prominent under conditions of high homeostatic sleep pressure (dark/active period and after sleep deprivation) and for the higher delta band (delta 2). Absolute spectral power provides information related to vigilance state quality and can also reveal differences in the microarchitecture of the cerebral cortex. Since elevated homeostatic sleep pressure is associated with higher synchrony in cortical neuron firing patterns [72, 73], it is tempting to speculate that *Nlgn1* KO mice present a higher synchrony of neuronal firing in the delta range. This is also supported by findings of steeper slope and higher amplitude of individual SW during SWS, a phenotype also found in males lacking NLGN1 [27]. A steeper slope represents an indication of elevated synchrony between neurons in transitions from the OFF and ON states of neuronal firing characterizing SW, while a higher amplitude suggests that a larger population of neurons is active/inactive simultaneously [72, 74]. The observation of a strong effect on delta 2 activity, combined with data showing a notable contribution of thalamocortical circuits in driving a rebound in the delta 2 range [49], supports that changes in thalamocortical connectivity due to the constitutive absence of NLGN1 contribute to modifications in SW properties. Nevertheless, the relative 24-hour variations in delta activity in KO mice was less impacted when considering delta 2, which could indicate modifications in mechanisms linked to SW generation rather than in their daily dynamics.

Regarding arrhythmic activity, *Nlgn1* KO females displayed the most prominent effect during SWS (baseline and REC) with a higher *Hm* and an amplified 24-hour dynamics of *Dispersion*. This can be interpreted as a generally higher self-similarity/persistence (lower complexity) across time scales and a changing level of ECoG multifractality/local instability, respectively. Indeed, in the absence of NLGN1, *Dispersion* becomes more negative (more unstable/multifractal) at the beginning of the dark periods and following sleep deprivation, while it reaches its less negative value (less unstable/multifractal) at the end of the light periods. This suggests that the across-scale organization of network activity, specifically during SWS, is controlled by NLGN1 in a manner that depends on wake/sleep history. This could be linked to the role of NLGN1 in shaping the excitation/inhibition ratio of the cerebral cortex [13], since this ratio was proposed to contribute to arrhythmic activity of the ECoG [75, 76]. Changes in arrhythmic electrophysiological activity are particularly relevant to inform about alterations in cognitive processing, such as working memory performance [77], discourse comprehension [78], and global cognitive scores in patients with neurodegenerative diseases [79]. Interestingly, these findings in females differ from previous observations in males, in which the KO of *Nlgn1* did not affect multifractal activity during SWS but triggered more anti-persistence (decreased *Hm*) during wakefulness [30]. This provides further support to the requirement for investigating effects of genetic manipulations in both biological sexes.

## Limitations

Although parallels have been drawn between the effects of the KO of *Nlgn1* in females and previous reports in males [26, 27, 30], it is important to note that direct comparisons cannot be made. Indeed, females and males were from different experimental cohorts studied years apart, and the genetic background of the present female mice is pure C57BL/6 (B6; 10-generation backcross), whereas males were of a mixed B6 and 129 Sv background. Since females and males share the B6 background and that wake/sleep phenotypes are generally similar between B6 and 129 strains [47, 48, 80], we anticipate that the different observations in females and males are nevertheless mainly linked to biological sex. In addition, the phase of the estrous cycle, which impacts wake/sleep phenotypes such as the time spent in vigilance states and the response to sleep deprivation [32, 38], was not controlled for in the present investigation. For instance, in rats, proestrus is generally associated with more time spent in wakefulness, less time spent in SWS and PS, and more SWS delta power during the dark period in comparison to other phases of the cycle [38, 81]. In mice, only less time spent in PS measured during the dark period was reported in proestrus (no change in other states or in SWS delta dynamics) [82]. It is thus possible that additional differences in wake/sleep architecture and in ECoG activity in *Nlgn1* mutant females can only be revealed when comparing genotypes at specific phases of the estrous cycle, which should be examined in future studies evaluating the role of NLGN in wake/sleep regulation in females. Lastly, the waking behavior of female *Nlgn1* KO mice was not interrogated in this study, and it is therefore not possible to assess its potential relationship to wake/sleep phenotypes, including fragmentation observed after sleep deprivation. Behavioral and cognitive alterations in male *Nlgn1* KO mice are subtle and often absent [16, 83], are generally similar in *Nlgn1* KO females [83], and involve a decreased preference for social novelty [26, 83]. Locomotor activity, as assessed using running-wheels under a light-dark cycle or beam breaks, was also shown to be preserved in mice of both sexes lacking NLGN1 [Srikanta, Ballester Roig et al., in preparation; [83]. Therefore, it is not anticipated that sex differences in behavior explain the apparent differences in wake/sleep phenotypes between female and male KO mice.

## Conclusion

In sum, *Nlgn1* KO females display an ensemble of alterations in wakefulness and sleep quality that differ in several ways from the changes we have previously reported in males (e.g., in females only: more time spent in PS, and arrhythmic activity differences solely in SWS). Knowing that clinical symptoms in patients diagnosed with the same disease can importantly differ between men and women, our study provides support to the importance of characterizing the effects of a genetic manipulation in both sexes, particularly in the context of neurodevelopmental/psychiatric disorders like autism spectrum disorders, for which mutations in NLGN have been reported. In autism spectrum disorders, studies have shown thalamocortical hypersynchrony along with excessive power in low-frequency bands [16, 84, 85]. Our related findings (e.g., elevated SWS delta activity and SW slope/amplitude in *Nlgn1* KO mice) thus suggest that NLGN1 is part of an ensemble of mechanisms explaining these alterations in patients.

## Methods

### Animals and protocol

*Nlgn1* KO mice were obtained from Jackson Laboratory (B6;129-*Nlgn1*^tm1Bros^/J) and backcrossed for more than 10 generations to C57BL/6J mice (Jackson Laboratory). The mutation results in an absence of NLGN1 protein due to the deletion of coding exons 1 and 2 as described previously [57]. Males and females heterozygous for the mutation were bred to obtain WT mice, heterozygous mice and *Nlgn1* KO homozygous mice. Animals were maintained under a 12-hour light/12-hour dark cycle and between 23 and 25 °C, with free access to food and water, throughout breeding and experimental procedures. Eleven WT (20.7 ± 1.1 g) and 13 KO (21.3 ± 1.5 g) female littermates have been studied here. Female mice were submitted to electrode implantation surgery as detailed below between 10 and 13 weeks of age. After surgery, mice recovered for about five days and adapted to cabling conditions for seven days before recordings. ECoG recordings started at light onset (defined as Zeitgeber time 0: ZT0) and lasted 48 hours including 24 hours of undisturbed/baseline conditions, a 6-hour sleep deprivation and 18 hours of undisturbed/recovery conditions (24-hour REC). Sleep deprivation was performed by approaching a pipette near the mouse only when it was showing signs of sleep (e.g., immobility, sleep posture). Sleep deprivation also involved moving the animal to a cage with clean litter at the middle of the 6-hour procedure, and delicate tissue was added to the cage during the third and sixth hours only if sleep was increasingly difficult to prevent. All efforts were made to minimize stress during the sleep deprivation. It should be noted that even if sleep deprivation increases corticosterone levels in mice, this increase was shown to have no impact on SWS rebound and delta power response [86].

### Electrode implantation surgery

*Nlgn1* KO mice and WT littermates were implanted with ECoG and electromyographic (EMG) electrodes as previously described [87, 88]. Briefly, surgeries were performed under Ketamine/Xylazine anesthesia (120/10 mg/kg, i.p. injection). ECoG electrodes consisted of two gold-plated screws that were screwed through the skull over the right cerebral hemisphere (anterior/motor cortex: 1.5 mm lateral to midline, 1.5 mm anterior to bregma; posterior/visual cortex: 1.5 mm lateral to midline, 1.0 mm anterior to lambda; see [89]). An additional screw was placed on the right hemisphere and served as a reference (2.6 mm lateral to midline, 0.7 mm posterior to bregma). Three anchor screws were implanted over the left hemisphere. Two gold wires inserted in neck muscles served as EMG electrodes. The ECoG and EMG electrodes were soldered to a connector and fixed to the skull with dental cement, along with the anchor screws.

### ECoG recording and analyses

ECoG/EMG were recorded, and signals were amplified (Lamont amplifiers), sampled at 256 Hz and filtered using the commercial software Harmonie (Natus, Middleton, WI) as done previously [24, 87, 88]. Vigilance states (wakefulness, SWS and PS) were determined by visual inspection of bipolar signals and assigned to 4-second epochs, based on ECoG and EMG characteristics, as detailed previously [47, 90]. Briefly, wakefulness was identified by low amplitude ECoG signal with mixed frequencies together with high amplitude and variable EMG signal. SWS was identified by high amplitude ECoG signal, with little activity at the EMG signal level. PS was characterized by regular theta waves (6-9 Hz) on the ECoG signal with the lowest EMG tone. Artifacts were simultaneously identified and later excluded for spectral analysis. Vigilance state amounts were calculated for the 12-hour light and 12-hour dark periods and expressed as a percentage of total recording time for both baseline and REC. Time spent in vigilance state (in minutes) and the number of short bouts for wakefulness (≤ 16 seconds), SWS (≤ 32 seconds) and PS (≤ 16 seconds) were computed for each hour of the 48-hour recording. Upper limits for short episodes were determined from previous literature [46, 47, 49, 91]. The mean bout duration of individual state episodes was averaged for the 12-hour light and 12-hour dark periods in baseline and REC. For REC, the total time spent in SWS during sleep deprivation was computed. In addition, the latency to SWS following sleep deprivation was calculated as the time elapsed from the end of sleep deprivation to the first bout of SWS lasting at least 60 seconds and not interrupted by more than two 4-second epochs of wakefulness. Likewise, the latency to PS was defined as the elapsed time between the end of sleep deprivation and the first 4-second epoch scored as PS [70]. Latency difference was calculated by subtracting SWS latency from PS latency. The effect of sleep deprivation and recovery on SWS and PS duration was assessed using accumulated differences from the corresponding baseline values. Finally, the number of bouts of different durations was calculated for every state for the full 24-hour of baseline and REC.

### Spectral analysis of the ECoG

The bipolar ECoG signal of 4-second epochs without artifact was subjected to a Fast Fourier transform to compute spectral power between 0.75 and 50 Hz (0.25 Hz resolution; graphed between 0.75 and 30 Hz to facilitate visualization of between-genotype differences). To account for interindividual differences in overall ECoG power, ECoG spectra were expressed as a percentage of the mean total ECoG power across all frequencies of all states during baseline for each individual mouse. Only epochs visually identified as quiet wake (low amplitude EMG) were used for wake spectral analyses to ensure no contamination of the ECoG by EMG activity. Spectral activity was also computed separately for delta (1-4 Hz), delta 1 (0.75-1.75 Hz), and delta 2 (2.5-3.5 Hz) frequency bands during SWS. To take into account the distribution of sleep and wakefulness, the activity in these spectral bands was averaged per interval each comprising a similar number of SWS epochs within an animal as done previously [87, 88]. More precisely, during the 24-hour baseline, spectral power was calculated for 12 equal intervals during the light period and for six equal intervals during the dark period. For recovery, spectral power was calculated for eight equal intervals during the light period after the end of sleep deprivation and for six equal intervals during the dark period. The dynamics was calculated for relative power in spectral bands, which was computed by expressing the power of each interval of baseline and REC as a percentage of the mean value of the last four hours of the baseline light period (ZT8 to 12, which corresponds to the minimal level of SWS delta activity over the nychthemeron). The theta/delta activity ratio was calculated for PS for each animal by dividing the 24-hour mean of absolute spectral power in theta frequencies (6-9 Hz) by the 24-hour mean of absolute spectral power in delta frequencies (1-4 Hz) separately for baseline and REC.

### Detection and analyses of SW

Detection of individual SW was performed on the bipolar ECoG signal of SWS artifact-free epochs after band-pass filtering (between 0.5 and 4 Hz) using a linear phase FIR filter (-3 dB). The following criteria were used to detect SW (see also [25, 27, 51]): negative-to-positive peak-to-peak amplitude > 120 µV, negative peak amplitude > 40 µV, negative phase duration between 0.1 and 1.0 second and positive phase duration < 1.0 second. For each SW, four parameters were derived: peak-to-peak amplitude (difference in voltage between the negative and positive peaks of unfiltered signal [µV]), slope (velocity of the change between the negative and positive peaks [µV/second]), duration of the negative phase (second), duration of the positive phase (second). SW parameters were computed for the 12-hour light and 12-hour dark periods during baseline and REC. To assess their 24-hour dynamics, parameters were also averaged for the same intervals described above for the activity in the different delta bands. To further investigate the effect of the KO on the homeostatic-dependent dynamics of SW parameters, a decay was calculated during initial REC hours using the difference between the last and first intervals of the first 6-hour recovery following sleep deprivation.

### Multifractal analysis of the ECoG

A multifractal analysis was conducted to evaluate the scaling properties and regularities/irregularities across time scales of the ECoG as previously done [30, 92]. This approach represents the scale invariance of the ECoG signal by 1/f^γ^, which is observed as the slope of the power decay in a log-log plot of spectral power and frequency (frequency range 0.5 to 64 Hz). Using a Wavelet-Leaders formalism developed according to previous methodologies [93, 94], the Hurst exponent (*H*) was obtained from the scaling exponent γ = 2*H* + 1 and a Daubechies wavelet with six vanishing moments. All possible *H* were extracted for each artifact-free 4-second epoch and the most prevalent *H* (*Hm*) for each epoch was used as a measure of “anti-persistence/complexity” across time scales, whereas the *Dispersion* of all *H* around *Hm* was used as an indicator of “multifractality”. The two metrics were extracted for the full 24-hour baseline and the full 24-hour REC, and their 24-hour dynamics was also interrogated using equal intervals of wakefulness and sleep (see above).

### Protein extraction and Western blot

Total protein was extracted from bilateral cerebral cortex regions dissected from brain frozen on dry ice approximately one week after ECoG/EMG recording similar to previously performed [95]. Briefly, brain regions were placed in ice-cold modified RIPA buffer (50 mM HEPES, 10 mM EDTA, 0.1% SDS, 1% IGEPAL, 0.5% sodium deoxycholate, 1 mM PMSF, protease and phosphatase inhibitors [Sigma-Aldrich, St. Louis, MO]) and homogenized on ice (PowerGen 125 homogenizer, Thermo Fisher Scientific, Montreal, Canada). Homogenates were centrifuged at 12,000 g for 10 minutes at 4 °C, and the supernatants (40 µg of protein per sample) was loaded on 8% polyacrylamide gels for migration/separation (100 Volts for 75 minutes). Proteins were then transferred to PVDF membranes (100 Volts for 90 minutes), and probed using the following antibodies: mouse anti-NLGN1 (1:1000; 129111, Synaptic Systems GmbH, Goettingen, Germany), mouse anti-Actin (1:3000; A5441, Sigma-Aldrich), and HRP-conjugated donkey anti-mouse (1:1000; sc-2318, Santa Cruz Biotechnology, Dallas, TX). The proteins were then revealed by incubating membranes with Clarity Max ECL solution (Bio-Rad Laboratories, Hercules, CA), and visualized using a ChemiDoc imager (Bio-Rad Laboratories).

### Statistical analyses

Statistica 6.1 (StatSoft Inc., Tulsa, OK) was used to perform statistical analyses and Prism 10.2 (GraphPad Software Inc., La Jolla, CA) to produce figures. SWS during sleep deprivation, sleep latencies and decay of SW properties were compared between genotypes (WT vs. KO) by Student’s t-tests. Differences in vigilance state duration per light/dark periods, per hour, in bout number per hour, in bouts of different durations, and in time courses of delta, delta 1, delta 2, SW and multifractal parameters were assessed for baseline and REC using two-way repeated-measures analyses of variance (ANOVA; with factors Genotype, Period, Hour, Duration, and Interval). Differences in theta/delta activity ratio, SW and multifractal parameters per condition (i.e., baseline versus REC) were assessed using two-way ANOVAs with factors Genotype and Condition. For Fig. 1C, ANOVAs were performed including 24 hours in baseline and including 18 hours in REC given the lack of variance for hours of the sleep deprivation. A similar design was applied for PS of Fig. 2C. Significant effects were adjusted using the Huynh-Feldt correction and significant interactions were decomposed using planned comparisons or Student’s t-tests. The threshold for statistical significance was set to 0.05, and results are reported as mean ± SEM. Two animals (one WT and one KO) were removed from statistical analyses for the time course analyses of multifractal metrics only (Fig. 5B and D, and 6) because of outlier values found for two or more intervals during REC.

## Data Availability

All raw data will be made available on reasonable request.

## Abbreviations

ANOVA: Analysis of variance
BL: Baseline
ECoG: Electrocorticography
EMG: Electromyography
h: Hour
*H*: Hurst exponent
*Hm*: Most prominent Hurst exponent
KO: Knockout
min: Minute
NLGN: Neuroligin
NLGN1: Neuroligin-1
NMDA: N-methyl-D-aspartate
PS: Paradoxical sleep
REC: Recovery
sec: Second
SD: Sleep deprivation
SEM: Standard error of the mean
SW: Slow waves
SWS: Slow wave sleep
WT: Wild-type
ZT: Zeitgeber time
